# A comprehensive system for eyelid analysis using deep learning: automated measurement of eyelid position and corneal exposure

**DOI:** 10.1007/s13534-025-00546-9

**Published:** 2026-01-09

**Authors:** Bokeun Song, Yeon Gyu Han, Sundariya Tsetsegjargal, Kyungmin Cho, Chang-Wook Seo, Sunje Kim, Dongheon Lee

**Affiliations:** 1 Department of Plastic and Reconstructive Surgery, College of Medicine, Chungnam National University, Daejeon, Republic of Korea; 2 Department of Plastic and Reconstructive Surgery, Chungnam National University Hospital, Daejeon, Republic of Korea; 3 Department of Biomedical Engineering, Chungnam National University College of Medicine, Daejeon, Republic of Korea; 4 Anigma Technologies Inc., Daejeon, Republic of Korea; 5 Department of Radiology, Seoul National University College of Medicine, Seoul National University Hospital, Seoul, Republic of Korea; 6 Institute of Medical and Biological Engineering, Seoul National University Medical Research Center, Seoul, Republic of Korea; 7 Interdisciplinary Program in Artificial Intelligence, Seoul National University, Seoul, Republic of Korea

**Keywords:** Eyelid analysis, Marginal reflex distance, Corneal exposure ratio, Infrared imaging, Deep learning

## Abstract

Quantitative eyelid analysis is crucial for diagnosing and managing eyelid disorders, such as ptosis, and for surgical planning. However, traditional manual measurements of Marginal Reflex Distance (MRD) and Corneal Exposure Ratio are often error-prone and inconsistent. Existing deep learning approaches using RGB imaging face challenges in accurately delineating eye structures due to insufficient contrast and varying iris pigmentation. This study introduces a comprehensive system integrating infrared (IR) imaging with deep learning-based eye segmentation to overcome these limitations. The system utilizes an IR camera with facial rotation compensation for consistent image acquisition and employs deep learning algorithms optimized for IR imaging characteristics. A dataset of 2,121 IR images from 303 patients (internal validation) and 406 public dataset images (external validation) was used. Multiple models including RITnet and SegFormer were evaluated, with data augmentation techniques applied to improve performance. SegFormer with data augmentation demonstrated the highest performance, achieving mean IoU of 0.9325 for internal validation and 0.9288 for external validation. Our automated system achieved improved precision compared to manual measurements, with MRD1 MSE of 0.362±0.511 mm and MRD2 MSE of 0.286±0.324 mm. This proposed system offers a robust, efficient, and standardized approach for quantitative eyelid analysis, leveraging IR imaging advantages to address the shortcomings of conventional assessment methods and providing clinical-grade accuracy for automated eyelid parameter measurement.

**Supplementary Information:**

The online version contains supplementary material available at 10.1007/s13534-025-00546-9.

## Introduction

Eyelid shape and contour play a critical role not only in aesthetics but also in diagnosing and managing various medical conditions [1]. Comprehensive eyelid analysis is a fundamental aspect of ophthalmic and oculoplastic evaluations, offering valuable insights into both functional and aesthetic aspects of the periorbital region [2]. Precise assessment of eyelid parameters is essential for diagnosing disorders such as ptosis, eyelid retraction, and thyroid eye disease, as well as for planning and evaluating surgical outcomes [3]. Among these conditions, blepharoptosis alone affects a substantial portion of the adult population, with prevalence rates ranging from 11.5% to 13.5% in general populations and increasing dramatically with age [1]. These conditions significantly impact patients’ quality of life through functional visual impairments, compensatory postural changes, and psychosocial effects, making accurate measurement protocols critical for optimal patient care and successful therapeutic interventions.

Quantitative eyelid analysis provides objective and measurable data, which significantly enhances diagnostic accuracy and surgical planning compared to subjective visual assessment methods that have historically dominated clinical practice. Key parameters, such as Marginal Reflex Distance (MRD) and Corneal Exposure Ratio (CER) are indispensable for evaluating eyelid function, symmetry, and postoperative outcomes [4–6]. The clinical significance of MRD measurements, particularly MRD1 for upper eyelid assessment, has been well-established as the gold standard for ptosis evaluation since Putterman’s seminal work [4]. However, traditional manual methods for measuring these parameters suffer from substantial limitations, including low repeatability, significant inter-observer variability, user dependency, and susceptibility to human error. Studies have documented considerable measurement variations between experienced clinicians, with differences that can directly influence surgical decision-making and patient outcomes [5]. The clinical impact of these measurement inconsistencies extends beyond diagnostic uncertainty to affect surgical planning decisions, postoperative evaluation accuracy, and ultimately patient satisfaction, underscoring the critical need for precise, objective, and automated measurement approaches.

Previous studies have proposed various deep learning-based image processing techniques for pupil segmentation in quantitative eyelid analysis [7–13], with recent advances demonstrating automated eyelid parameter measurement from clinical photographs [28, 31–33]. However, these approaches have been primarily developed and tested under controlled laboratory conditions using RGB imaging modalities. Under RGB lighting conditions, the contrast between the dark iris and the similarly dark pupil is insufficient, making it challenging to delineate their boundaries accurately [14]. Additionally, iris pigmentation varies widely among populations; for example, darker or brown irises, commonly found in East Asian populations, further complicate precise measurements [8]. These technical challenges limit the reliability and clinical applicability of RGB-based automated measurement systems, particularly in diverse patient populations and varying clinical environments. The translation gap between laboratory performance and real-world clinical deployment has hindered widespread adoption of these automated systems in routine practice. To address these fundamental limitations, prior research has demonstrated that infrared (IR) light sources can improve the visual contrast of the pupil region and mitigate the effects of iris pigmentation, enabling more accurate segmentation of the pupil [15–17].

The fundamental advantage of infrared imaging stems from differential tissue optical properties at near-infrared wavelengths, which eliminate ambient lighting variations and provide enhanced pupil-iris contrast ratios regardless of iris pigmentation [8, 15]. This technological approach offers consistent imaging conditions independent of external lighting environments, a critical requirement for standardized clinical measurements across different healthcare settings. Unlike RGB imaging, infrared technology provides stable contrast characteristics that are minimally affected by individual anatomical variations in iris pigmentation or ambient lighting conditions [8]. The maturation and clinical validation of infrared-based imaging technology is evidenced by the universal adoption of infrared-based systems in commercial eye-tracking devices and clinical ophthalmic instruments [16–19]. However, despite these technological advances, existing IR-based systems have primarily focused on isolated measurement tasks and have not been integrated into comprehensive eyelid analysis platforms suitable for routine clinical use.

**Fig. 1:**
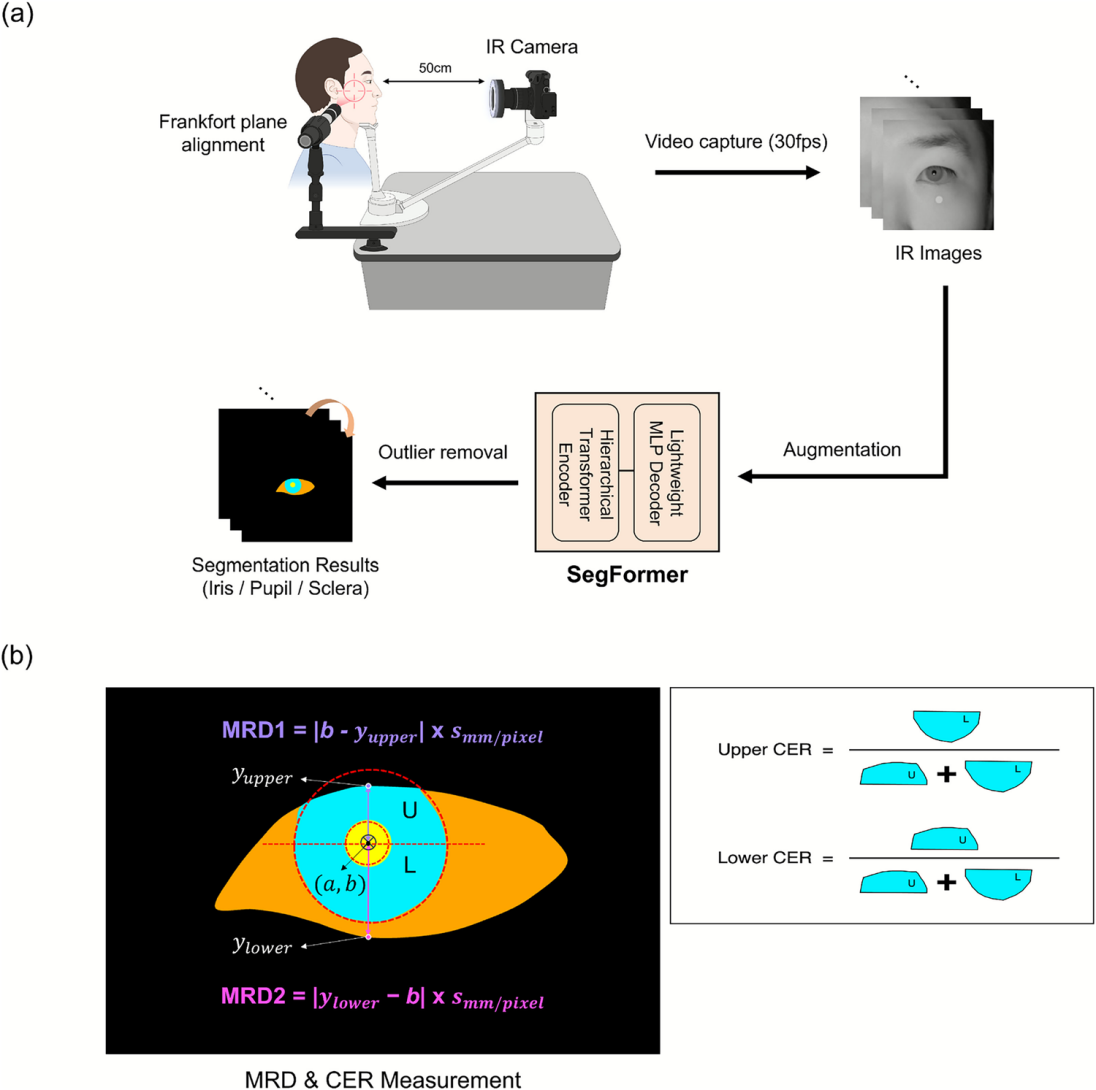
An overview of the comprehensive eyelid analysis system. **a** IR imaging device with Frankfort plane alignment and laser guidance (50 cm distance, 30 fps) and deep learning-based segmentation pipeline using SegFormer with data augmentation and outlier removal. **b** Automated MRD and CER measurement using circle regression and geometric calculations

In this study, we propose a comprehensive system for quantitative eyelid analysis that addresses the limitations of existing measurement approaches through integrated hardware and software innovations. While our prior feasibility study demonstrated the potential of IR imaging for MRD1 measurement [8], the current work extends this to a complete clinical platform that simultaneously quantifies multiple eyelid parameters (MRD1, MRD2, upper/lower CER) through integrated hardware-software design and validates clinical-grade accuracy via dual-center evaluation. This system incorporates an IR imaging camera with a head positioning guide to standardize image acquisition, addressing the positioning variability that can limit manual measurement techniques. Additionally, it employs a deep learning-based segmentation algorithm specifically optimized for infrared imaging characteristics. Our approach represents a departure from previous studies by combining infrared optical advantages with advanced deep learning in a unified clinical platform. By achieving measurements independent of operator variability and environmental conditions, our system enables automated extraction of MRD and CER parameters with clinical-grade accuracy, providing standardized results for enhanced diagnostic consistency.

## Methods

### System overview

The proposed comprehensive system is divided into two main components: the imaging device and an algorithm for eyelid analysis (Fig. 1). Images captured by the infrared (IR) camera are processed using a deep learning-based eye segmentation model to segment the eye regions. Based on these results, MRD and CER are quantitatively measured, with robust measurements ensured through laser guidance and outlier removal algorithms integrated into the imaging device.

This study employed a systematic approach combining hardware optimization, algorithmic development, and clinical validation. The research design prioritized integration of established infrared imaging technology with optimized deep learning algorithms, focusing on clinical translation rather than re-investigation of established optical principles.

This study adhered to the principles of the Declaration of Helsinki. Informed consent was obtained for the publication of patients’ images both online and in print media. All patients provided written informed consent, including consent for possible publication in online and print media. This study received approval from the Ethics Committee of Chungnam National University Hospital, Daejeon, Korea (CNUH-IRB No. 2023-06-098).

### Configuration of imaging device

**Fig. 2:**
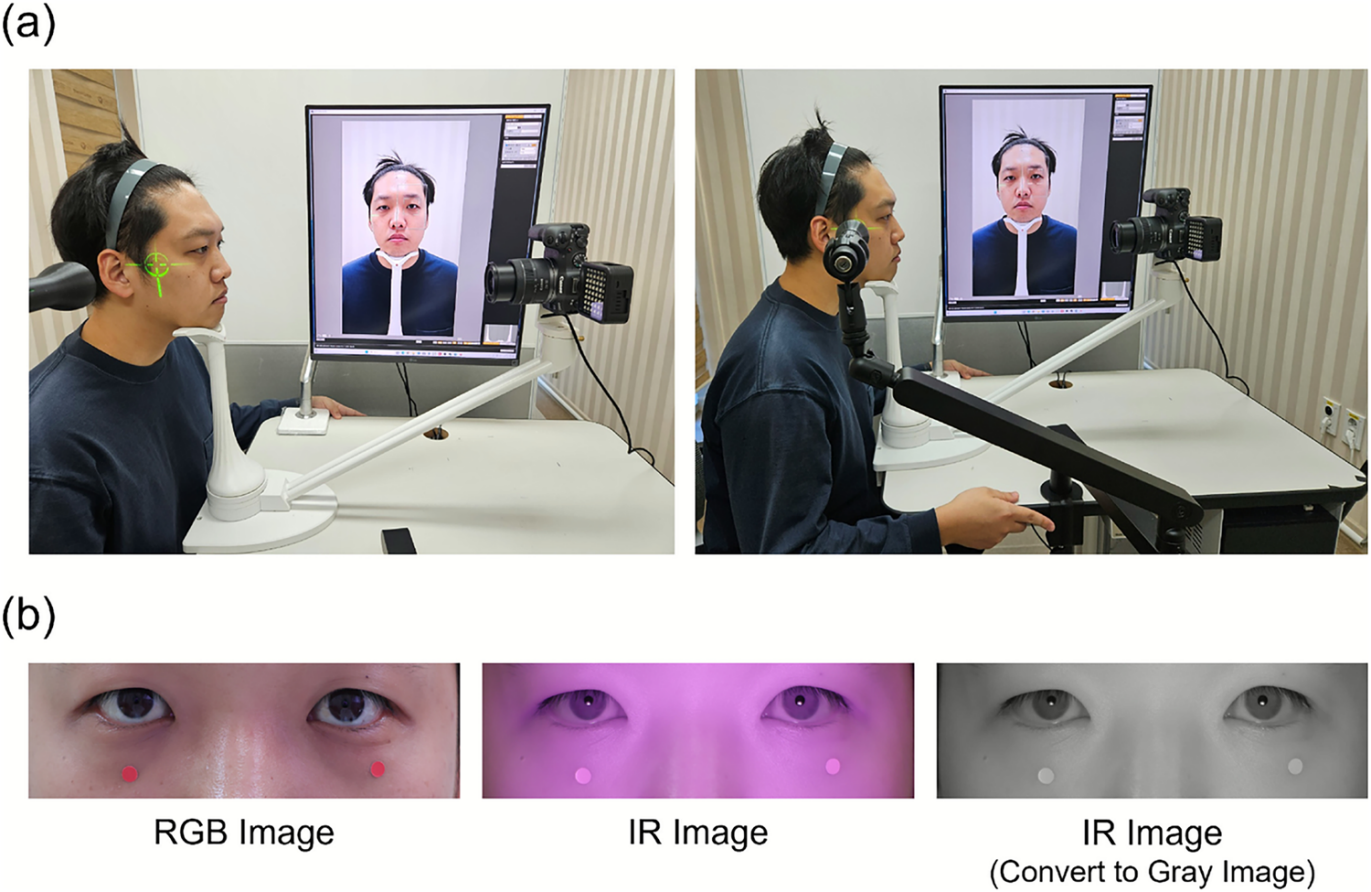
System Configuration and Captured Eye Images. **a** Configuration of Imaging Device. An Infrared (IR) camera is positioned at the front, and a cross-shaped laser on the side is used for face alignment. The desktop PC is equipped with software that handles eye segmentation and measurements. **b** Comparison of eye images captured in RGB, captured in infrared (IR), and the IR image converted to grayscale for identifying the pupil region

The imaging device utilized an industrial IR camera with near-infrared LED illumination (MS-IR510U; peak wavelength 850 nm, spectral width  40 nm; radiant intensity 85–110 mW/sr) to measure pupil position (Fig. 2b). Subjects were stabilized using a chinrest with forehead support at 50 cm, and a lateral cross-shaped laser ensured Frankfort-plane alignment to minimize sagittal-tilt bias [20]. Images were acquired at 30 fps and down-sampled to 256$\times$256 pixels for deep learning inference on a desktop PC (3.70 GHz CPU) (Fig. 2a). Pixel-to-millimeter scaling was obtained using 5 mm fiducial markers (session-wise median) without geometric camera calibration. Operation remained within IEC 62471 photobiological exposure limits; complete specifications are in Table S1.

A standard camera was modified to capture infrared images by removing the built-in infrared cut filter and substituting it with an infrared pass filter, thereby enabling the sensor to detect infrared light. To minimize facial movements during the examination, a facial support with a chin rest was employed, and the camera was positioned approximately 50 cm away from the patient’s eye level for capturing the image.

### Dataset and experimental setup

#### Data collection

This prospective observational study included 2,121 infrared images extracted from video recordings of 303 patients aged over 18 years who visited Chungnam National University Hospital between January 1, 2023, and December 30, 2023. Patient demographics, including age and sex, were recorded as shown in Table 1. Additionally, 406 images were obtained from 58 participants in the publicly available “Eye Movement Video Data” provided by AI Hub [21]. All images were systematically selected from video sequences, with frames containing closed eyes excluded to ensure only images suitable for eyelid analysis were utilized.

Three types of annotation data were used for eye segmentation: the background region, the iris region, the pupil region, and the sclera region, as illustrated in Fig. 1. The center coordinates of the pupil were also marked within a normalized coordinate system (pixel coordinates scaled to [0,1] range) to ensure precise localization. Two board-certified plastic surgeons performed the annotations using ImageJ (ver. 1.51). Inter-observer verification was conducted to ensure accuracy, and any discrepancies identified during validation were resolved through discussion.

#### Dataset preparation

We divided the dataset into training, tuning, and internal validation sets based on individual IDs to ensure that images of the same individual did not appear in both training and validation sets. The dataset consisted of a total of 2,527 images, with 1,365 images allocated to the training set, 329 images to the tuning set, and 427 images to the internal validation set from Chungnam National University Hospital. Additionally, 406 images from a public dataset were allocated to the external validation set.

**Table 1:** Dataset composition and demographics. Values are presented as number (%) or median [interquartile range]

Demographics	CNUH dataset			Public dataset
	Training set	Tuning set	Int val. set	Ext val. set
Number of Patients	195	47	61	58
Number of eyes	390	94	122	116
Number of Images	1,365	329	427	406
Demographics				
Age (years)	57.0 [44.0;62.0]	58.0 [51.0;63.0]	57.0 [47.0;62.0]	49.0 [39.0;59.0]
Sex				
Male	49 (25.0%)	12 (26.1%)	18 (29.3%)	28 (48.3%)
Female	146 (75.0%)	35 (73.9%)	43 (70.7%)	30 (51.7%)

Int val, Internal validation; Ext val, External validation

#### Preprocessing and data augmentation

All input images were resized to 256 $\times$ 256 pixels and preprocessed using gamma correction ($\gamma = 0.7$) to enhance pupil boundary contrast. Pixel values were normalized to [0, 1] and standardized to zero mean and unit variance.

To address the specific requirements of automated eyelid measurement while maintaining clinical accuracy, we applied data augmentation with carefully selected parameters. Unlike standard computer vision applications, clinical measurements require sub-millimeter precision, necessitating conservative transformation ranges.

Using the Albumentations package (ver. 2.0.1) [22], we applied the following transformations:

*Geometric Transformations:* Geometric transformations were applied to augment the training data while preserving the anatomical relationships essential for accurate MRD and CER measurements. The transformation parameters were set to conservative ranges compared to conventional computer vision applications: rotation angles were limited to $\theta \in [-3^\circ , 3^\circ ]$ (versus typical $\theta \in [-15^\circ , 15^\circ ]$), translation offsets to $(\Delta x, \Delta y) \in [-5\%, 5\%]$, and scaling factors to $s \in [0.9, 1.1]$ (versus typical $s \in [0.5, 2.0]$). These constrained ranges ensure that the spatial relationships between eyelid landmarks remain anatomically plausible. The geometric transformation set is defined as:

1 $$\begin{aligned} \mathcal {T}_{geo} = \{T_r(\theta ), T_t(\Delta x, \Delta y), T_s(s)\} \end{aligned}$$

where $T_r$, $T_t$, and $T_s$ represent rotation, translation, and scaling transformations, respectively.

*Photometric Adjustments:* Brightness and contrast modifications were applied within $\pm 15\%$ range, with gamma correction $\gamma \in [0.6, 0.9]$. These conservative ranges maintain the enhanced pupil-sclera contrast provided by IR imaging while introducing sufficient variation for model robustness.

*Additional Techniques:* We applied Gaussian blur ($\sigma \le 1.0$), random cropping (85-100% with aspect ratio preservation), and mild elastic deformation to simulate natural imaging variations while preserving eyelid boundary characteristics essential for precise measurement.

The combination of these conservative augmentation parameters resulted in improved model generalization while maintaining measurement consistency within clinical tolerance ranges.

### Deep learning-based segmentation approach

#### RITnet

We used the Real-time Iris Tracking Network (RITnet) as the baseline model, which is designed for real-time segmentation of the iris and pupil [23]. RITnet combines U-Net [24] and DenseNet [25] architectures to accurately segment the iris and pupil regions in images. Its encoder, based on ResNet [16], extracts features at multiple levels, while the decoder, built on DeconvNet [17], utilizes these features to produce high-resolution segmentation masks. Additionally, RITnet is lightweight, employing Depthwise Separable Convolution [26] to minimize the number of parameters and layers, thereby ensuring fast inference speeds.

#### SegFormer

We also used SegFormer as a baseline model, employs an encoder-decoder architecture in which the encoder divides the input image into patches and extracts features using Transformer blocks [27]. These blocks leverage multi-head self-attention to enable interactions between patches, capturing the overall structure and relationships within the image. The decoder then upsamples these features to generate high-resolution segmentation masks. By combining Transformer blocks with convolutional layers, SegFormer efficiently processes both global and local information, achieving faster inference speeds compared to traditional CNN-based models.

#### Implementation details

Python (version 3.8.19) was used for implementation. The baseline models were implemented using PyTorch (version 2.2.2) and trained on an NVIDIA GeForce RTX 3090 GPU. The batch size was set to 4, and the initial learning rate was set to 1e-4. The Adam optimizer was used for optimization, and the model was trained for a total of 150 epochs. Cross-entropy loss and dice loss were used as loss functions, and the final loss value was calculated by assigning weights to the two loss functions.

### Measurement and analysis framework

#### MRD and CER calculation

Based on the baseline model’s segmentation of the eye regions, we measured the Marginal Reflex Distance (MRD) and Corneal Exposure Ratio (CER) (Fig. 1). To account for cases where the iris is partially covered by the upper eyelid, the occluded area was estimated by assuming the iris to be circular and applying a circle regression method, as described in (2).

2 $$\begin{aligned} d_i = \sqrt{(x_i - a)^2 + (y_i - b)^2} - R \end{aligned}$$

where $(x_i, y_i)$ represents the coordinates of the *i*-th data point in a two-dimensional plane, while (*a*, *b*) denotes the coordinates of the center of the estimated circle. The parameter *R* corresponds to the estimated radius of the circle. The term $d_i$ represents the residual error, which quantifies the deviation of the given data point from the estimated circle. Specifically, it measures the difference between the actual Euclidean distance from the data point to the circle’s center and the estimated radius *R*. This formulation provides a geometric representation of the error in fitting a circle to the given data points, ensuring that the regression process aims to minimize these residuals to achieve an optimal estimation of the circle’s parameters.

**Eyelid Margin Extraction:** Before computing the MRD, the vertical coordinates of the upper and lower eyelid margins ($y_{upper}$ and $y_{lower}$) were algorithmically determined from the segmentation masks (Fig. 1b). Specifically, along the vertical line passing through the estimated pupil center (*a*, *b*), $y_{upper}$ was defined as the highest pixel coordinate of the segmented sclera or iris region. Conversely, $y_{lower}$ was defined as the lowest pixel coordinate of the segmented sclera or iris region. These coordinates represent the interface between the eyelid and the visible portion of the eyeball, measured along the vertical line passing through the pupil center.

Based on the estimated pupil center (*a*, *b*) and radius *R*, the clinical measurements are computed as follows:

**Marginal Reflex Distance (MRD):**

3 $$\begin{aligned} \text {MRD1}&= |b - y_{upper}| \times s_{mm/pixel} \end{aligned}$$

4 $$\begin{aligned} \text {MRD2}&= |y_{lower} - b| \times s_{mm/pixel} \end{aligned}$$

where $y_{upper}$ and $y_{lower}$ represent the vertical coordinates of upper and lower eyelid margins, respectively, and $s_{mm/pixel}$ is the pixel-to-millimeter conversion factor derived from the 5 mm reference markers.

*Corneal Exposure Ratio (CER):*

5 $$\begin{aligned} \text {CER}_{upper}&= \frac{\pi R^2 - A_{occluded,upper}}{\pi R^2} \end{aligned}$$

6 $$\begin{aligned} \text {CER}_{lower}&= \frac{\pi R^2 - A_{occluded,lower}}{\pi R^2} \end{aligned}$$

where $A_{occluded,upper}$ and $A_{occluded,lower}$ represent the occluded corneal areas by upper and lower eyelids, respectively, calculated as circular segments using the chord length determined by eyelid boundary intersections with the fitted circle.

#### Outlier removal

From the video captured by the imaging device at 30 fps, two frames per second were sampled. An outlier removal process was applied to ensure stable performance of the eye segmentation and eyelid analysis algorithms, particularly in cases of eye movement or blinking.

From the video captured by the imaging device at 30 fps, two frames per second were sampled. To ensure stable performance of the eye segmentation and eyelid analysis algorithms, a process to remove outliers was necessary, particularly in cases of eye movement or blinking.

**Fig. 3:**
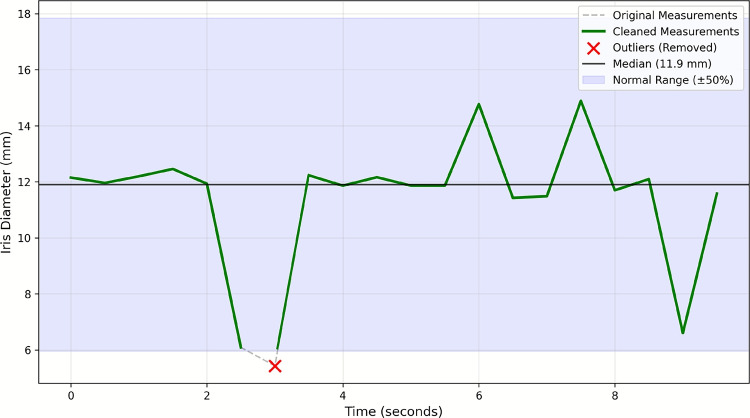
Example of the outlier removal process in iris diameter measurements. Original measurements (gray dashed line), cleaned measurements after outlier removal (green line), detected outliers marked with red X, median value (11.9 mm, horizontal line), and normal range (shaded area, ±50% from median) are shown. Time axis shows measurement sequence in seconds

The outlier detection algorithm employs statistical analysis based on the median absolute deviation (MAD) method for robust threshold determination:

7 $$\begin{aligned} \text {Outlier}_i = {\left\{ \begin{array}{ll} \text {True}, & \text {if } \frac{|m_i - \text {median}(\textbf{m})|}{\text {MAD}(\textbf{m})} > \tau \\ \text {False}, & \text {otherwise} \end{array}\right. } \end{aligned}$$

where $\textbf{m} = \{m_1, m_2, \ldots , m_n\}$ represents the measurement sequence, $\text {MAD}(\textbf{m}) = \text {median}(|m_i - \text {median}(\textbf{m})|)$ is the median absolute deviation, and $\tau = 2.0$ represents the threshold coefficient. Measurements identified as outliers by this criterion are excluded when they represent less than 50% of the existing measurements, ensuring sufficient data retention while minimizing distortion and enhancing reliability (Fig. 3).

### Performance evaluation

#### Evaluation metrics

We evaluated the baseline models’ segmentation results using the mean Intersection over Union (mIoU) for each of area within eye such as background, sclera, iris, and pupil. Additionally, we measured MRD and CER based on the segmentation results of the baseline models.

The MRD is divided into MRD1 and MRD2, and their values were evaluated using the Mean Square Error (MSE), calculated as the average squared difference between the predicted and actual MRD values. Similarly, CER is divided into upper CER and lower CER, which were also evaluated by calculating the average squared difference between the predicted and actual CER values.

The internal validation set was prepared with markers attached to the facial images. These markers were used as reference points for converting MRD and CER from pixels to millimeters, with the diameter of each marker being 5 mm.

#### Validation approach

The validation approach differed between MRD and CER measurements based on current clinical practice standards and measurement feasibility.

MRD measurements were validated against manual assessment performed by two board-certified plastic surgeons using established clinical protocols with penlight and ruler techniques. This approach reflects current clinical practice where MRD measurement has standardized procedures and documented inter-observer reliability in the literature.

CER measurements employed algorithmic validation comparing results across different deep learning models. This validation strategy was selected because CER quantification requires complex geometric calculations of corneal exposure ratios that are not performed manually in routine clinical practice. No standardized manual CER measurement protocols exist in current clinical workflows, making inter-algorithm consistency assessment the most appropriate validation approach for this computational metric.

For Bland-Altman analysis, frame-level manual annotations performed by board-certified surgeons using ImageJ (Methods 2.3.1) served as the primary reference. These annotations were measured from the same IR frames as automated predictions, satisfying Bland-Altman’s requirement for synchronized comparison. Clinical manual measurements from slit-lamp examination (Table 3) were available for Hospital dataset MRD1 and MRD2, and were compared separately to assess agreement under real-world clinical conditions.

#### Statistical analysis

The mean value of each MRD and CER measurement and the difference between them were analysed using a one-way analysis of variance (ANOVA) test. A box-plot graph was plotted to visually describe each different measurement. Bland-Altman plot analyses were performed to statistically examine the agreement between the different measurement methods. Statistical significance was set at $p<0.05$. Data obtained from the study were analysed using the Statistical Package for the Social Sciences version 26 (IBM Corporation, Armonk, NY, USA) and Microsoft Excel version 16.84 (Microsoft$\circledR$, Redmond, WA, USA).

## Results

### Comparative performance of eye segmentation models

We compare the performance of baseline models, RITnet [23], SegFormer [27] and SegFormer applied with several augmentation techniques. Table 2 shows the comparative performance of baseline models and SegFormer with augmentation approach demonstrated the highest performance. The highest performance was observed in the iris, sclera, and pupil regions, with statistical significance demonstrated in the internal validation set as 0.9325 and in the external validation set as 0.9288. Statistical significance was assessed using Wilcoxon signed-rank test, with *p*-values indicating comparison against SegFormer with augmentation techniques.

**Fig. 4:**
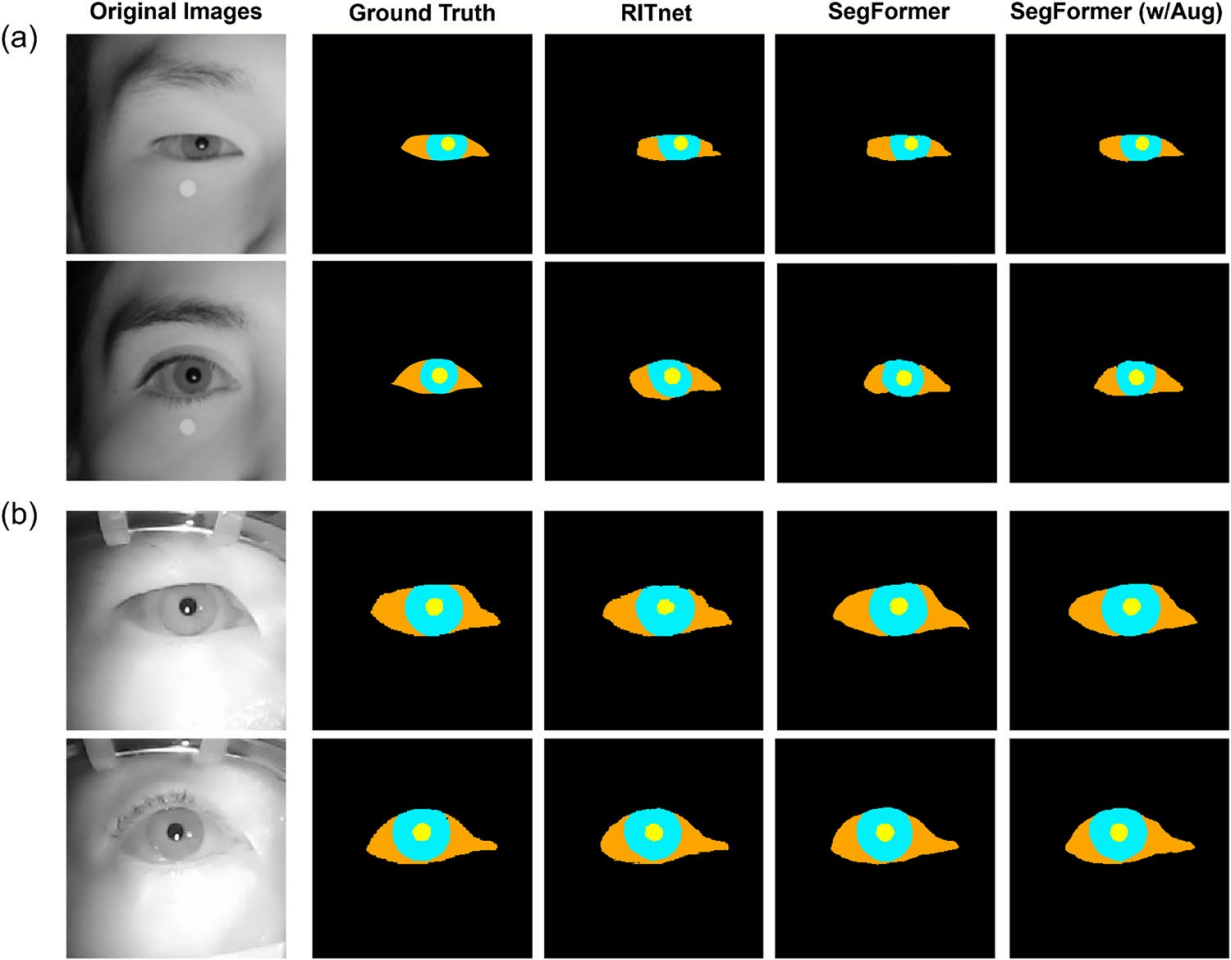
Quantitative results of eye segmentation models. **a** The results on internal validation set **b** The results on external validation set

Figure 4 represents the qualitative results of baseline models, showing the visual comparison of segmentation results across different models and validation datasets.

**Table 2:** Comparative performance of baseline models for eye segmentation

Model	Data type	mIoU					*p*-value
		Background	Iris	Sclera	Pupil	Average	(vs. C)
RITnet (A)	Int. val	0.9985	0.9224	0.8412	0.9223	0.9211	< 0.05
	Ext. val	0.9944	0.8938	0.8456	0.9241	0.9145	< 0.05
SegFormer (B)	Int. val	0.9985	0.9198	0.8448	0.9242	0.9218	< 0.05
	Ext. val	0.9950	0.8929	0.8621	0.9289	0.9197	< 0.05
SegFormer (w/ Aug.) (C)	Int. val	**0**.**9987**	**0**.**9411**	**0**.**8588**	**0**.**9313**	**0**.**9325**	–
	Ext. val	**0**.**9954**	**0**.**9088**	**0**.**8745**	**0**.**9364**	**0**.**9288**	–

Int val, Internal validation; Ext val, External validation; Bold values indicate the best performance.

### Quantitative results of MRD and CER

**Fig. 5:**
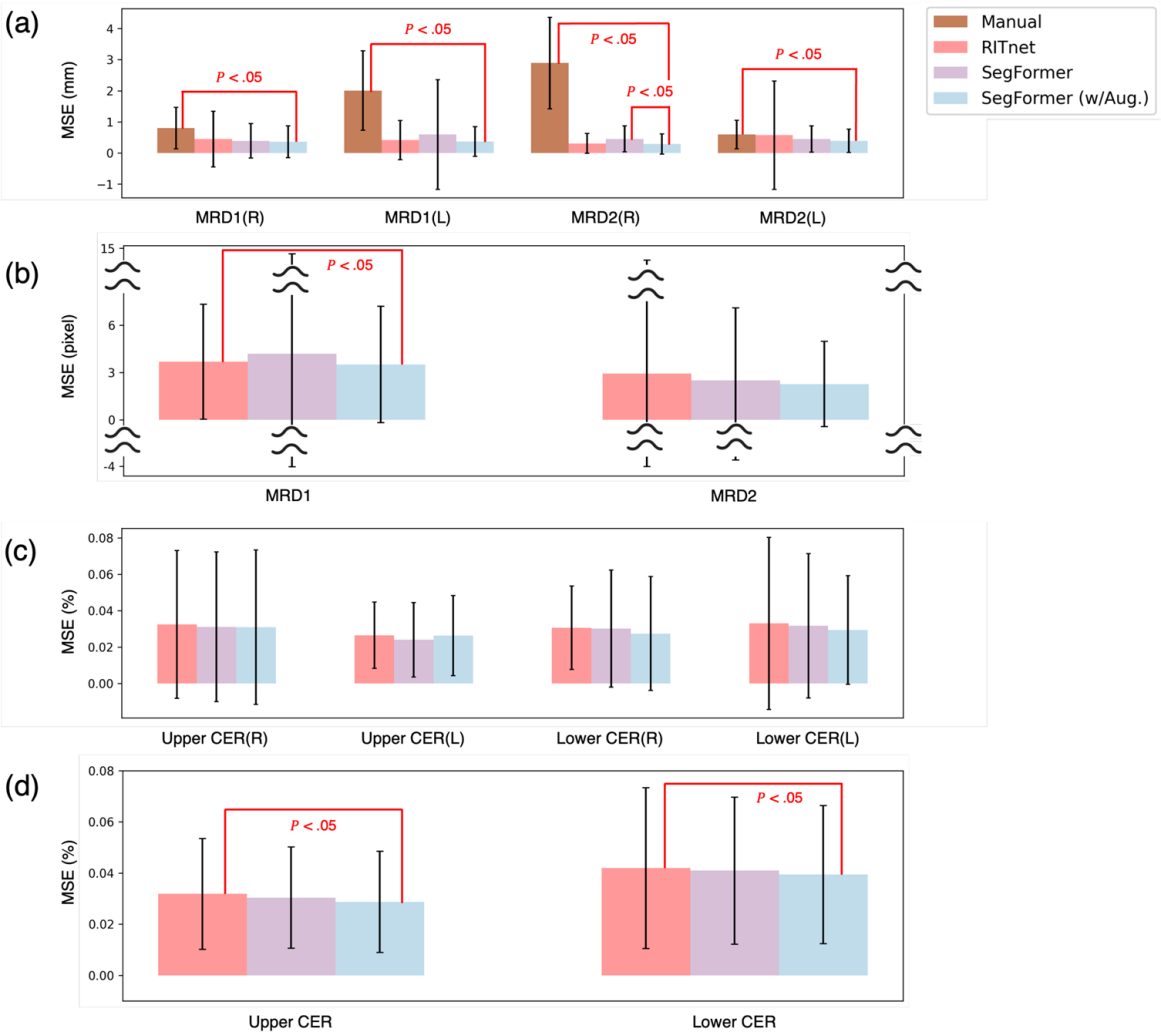
Quantitative results of MRD and CER measurements. Box plots show mean squared error (MSE) distributions for different methods (Manual, RITnet, SegFormer, SegFormer with augmentation). **a** MRD measurements from CNUH dataset (MSE in mm; R=right eye, L=left eye). **b** MRD measurements from public dataset (MSE in pixels). **c** CER measurements from CNUH dataset (MSE in %; upper and lower eyelid). **d** CER measurements from public dataset (MSE in %). Asterisks (*) indicate statistical significance (p < 0.05) compared to SegFormer with augmentation

Following the measurement validation approach described in Methods, MRD measurements were compared against manual assessment while CER measurements were evaluated through inter-algorithm comparison. Using the results from the best-performing eye segmentation model, SegFormer with augmentation, we measured MRD1, MRD2, as well as upper and lower CER, and calculated the corresponding errors. Figure 5 and Table 3 represents the quantitative results of MRD and CER measurements using box plots, along with relevant statistical analyses (Table 4).

**Table 3:** Comparative performance of MRD measurement

Dataset (Metric)	Manual	RITnet	SegFormer	SegFormer (w/ Aug.)
*CNUH dataset (MSE (mm))*				
MRD1(R)	0.799 ± 0.665[1]	0.446 ± 0.895	0.391 ± 0.551	**0.362 ± 0.511**
MRD1(L)	2.01 ± 1.277[1]	0.416 ± 0.628	0.596 ± 1.761	**0.37 ± 0.475**
MRD2(R)	2.89 ± 1.471[1]	0.31 ± 0.323	0.452 ± 0.418[1]	**0.286 ± 0.324**
MRD2(L)	0.593 ± 0.462[1]	0.572 ± 1.736	0.449 ± 0.42	**0.391 ± 0.371**
*Public dataset (MSE (pixel))*				
MRD1	–	3.683 ± 3.638[1]	4.196 ± 8.441	**3.508 ± 3.687**
MRD2	–	2.925 ± 7.047	2.505 ± 4.583	**2.275 ± 2.706**

MSE, mean squared error; MRD, marginal reflex distance. Values are presented as mean ± standard deviation. R, right eye; L, left eye. Statistically significant difference (p < 0.05) compared to SegFormer with augmentation; Bold values indicate the best performance.

**Table 4:** Comparative performance of CER measurement

Dataset (Metric)	Manual	RITnet	SegFormer	SegFormer (w/ Aug.)
*CNUH Dataset (MSE (%))*				
Upper CER (R)	–	0.033 ± 0.041	**0.031 ± 0.041**	0.031 ± 0.043
Upper CER (L)	–	0.027 ± 0.018	**0.024 ± 0.021**	0.026 ± 0.022
Lower CER (R)	–	0.031 ± 0.023	0.03 ± 0.032	**0.027 ± 0.031**
Lower CER (L)	–	0.033 ± 0.048	0.032 ± 0.04	**0.029 ± 0.03**
*Public Dataset (MSE (%))*				
Upper CER	–	0.032 ± 0.022[1]	0.03 ± 0.02	**0.029 ± 0.02**
Lower CER	–	0.042 ± 0.031[1]	0.041 ± 0.03	**0.039 ± 0.03**

MSE, mean squared error; CER, corneal exposure ratio. Values are presented as mean ± standard deviation. R, right eye; L, left eye. Statistically significant difference (p < 0.05) compared to SegFormer with augmentation; Bold values indicate the best performance.

**Fig. 6:**
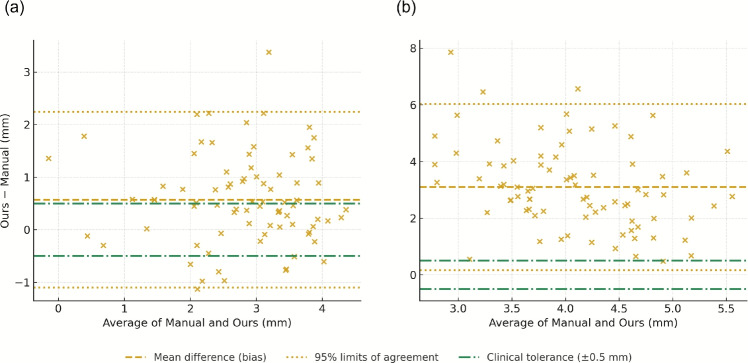
Bland-Altman analysis comparing automated and clinical manual measurements for MRD (Hospital dataset). **a** MRD1 (n=81, mean difference (bias) = 0.573 mm, 95% limits of agreement (LoA) = [$-$1.214, 2.360]). **b** MRD2 (n = 81, bias = 3.182 mm, 95% LoA =[0.195, 6.169]). Green dash-dot lines indicate clinical tolerance (±0.5 mm). Larger discrepancies compared to frame-level annotations (Fig. S1) reflect differences in measurement conditions and timing

Regarding MRD, the proposed method yielded the lowest error values compared to other methods. In particular, for MRD1—which is routinely measured in clinical practice using manual techniques with ruler and penlight—we performed comparisons using the CNUH dataset. The results showed a statistically significant difference between the manual method and the proposed method for both MRD1 and MRD2. Additionally, there was a statistically significant difference between the simple SegFormer model and the proposed method for right MRD2.

For the public dataset, left and right eyes were not separately labeled, and since no markers were available to serve as reference points, errors were measured in pixel units to maintain consistency. Notably, for MRD1 in the public dataset, there was a statistically significant difference compared to RITnet. Furthermore, for most CER measurements, the proposed method also achieved the lowest error values. In particular, in the public dataset, the proposed method showed a statistically significant difference from RITnet for both the upper and lower CER measurements.

Converting MSE to RMSE, our automated system achieved 0.6$-$0.7 mm for MRD measurements, comparable to reported inter-observer variability (0.5$-$1.0 mm) [5, 28] and significantly lower than manual measurements (Table 3, p < 0.05).

Bland-Altman analysis using frame-level manual annotations demonstrated excellent agreement (Figs. S1 and S2). For internal validation, MRD1 and MRD2 showed mean biases of 0.063 and 0.289 px with 95% limits of agreement (LoA) of [$-$1.259, 1.385] and [$-$0.779, 1.358] px. CER measurements showed mean biases < 0.04% and LoA < 0.1%.

Comparison with clinical manual measurements (Hospital dataset, Fig. 6) revealed MRD1 bias of 0.573 mm (LoA: [$-$1.214, 2.360] mm) and MRD2 bias of 3.182 mm (LoA: [0.195, 6.169] mm). The MRD1 bias approached clinical tolerance (±0.Altman analysis, frame-level manual 5 mm), while MRD2 showed substantial systematic offset.

## Discussion

This study introduces a comprehensive system for quantitative eyelid analysis, combining an imaging device with a deep learning-based algorithm. Unlike our previous feasibility study focused solely on MRD1 [8], this system provides simultaneous quantification of multiple clinical parameters essential for both surgical planning and outcome evaluation, integrated within a hardware-software framework designed for routine clinical use. The imaging device, equipped with an infrared camera, captures eye images and significantly enhances the accuracy of pupil segmentation. By employing this approach, the system achieves stable and precise measurements, facilitating the automated extraction of essential clinical parameters, including Marginal Reflex Distance (MRD) and Corneal Exposure Ratio (CER). Among the evaluated models, the SegFormer with augmentation approach achieved the best performance. Using this model, the measured MRD1, MRD2, as well as upper and lower CER, exhibited errors within clinically acceptable limits.

The clinical accuracy of our system warrants emphasis. Our RMSE values of 0.6-$-$0.7 mm for MRD measurements approach clinical tolerance (±0.5 mm) and reported inter-observer variability (0.5-$-$1.0 mm) [5, 28]. Our validation employed two measurement references. Frame-level annotations (Figs. S1, S2) demonstrated excellent agreement (mean biases < 0.3 px), confirming technical accuracy. Comparison with clinical manual measurements (Fig. 6) revealed larger discrepancies, particularly for MRD2 (bias = 3.182 mm), reflecting measurement timing and protocol differences, indicating that protocol standardization is needed before clinical interchangeability.

While MRD1 and CER are widely used for surgical outcome evaluation, they have limitations in capturing the dynamic interplay between upper and lower eyelid movements. Ptosis surgery affects both MRD1 and MRD2, altering corneal exposure in complex ways [28]. Dividing CER into upper and lower components enables a more detailed analysis of these changes. Integrating MRD1, MRD2, and separate CER metrics offers a comprehensive approach to evaluating surgical outcomes, improving precision in assessment, and understanding of eyelid dynamics [30].

In this study, while there were no significant performance differences among the baseline models used, it was confirmed that applying various augmentation techniques to the SegFormer model resulted in improved generalization ability, leading to statistically significant performance differences (Table 3). Additionally, to ensure accurate measurements of MRD and CER, IR images were captured, and techniques such as circle regression for precise localization of the pupil center, even when partially occluded by the eyelid, as well as outlier removal methods to address issues like blinking, were applied. These considerations reflect various efforts to adapt the proposed system for practical clinical use.

*Limitations and Future Works:* First, this study relies on surrogate markers for measurement, as the system defines MRD from the pupil center and CER from the iris boundary rather than the clinical gold standards of the corneal light reflex (glint) and the corneal limbus. While these markers provide high computational stability within our IR imaging context, this deviation represents a potential source of systematic bias, via the pupil–glint offset (chord $\mu$) on the order of tenths of a millimeter  [34]. Our laser-guided alignment minimizes this variability, and our achieved RMSE of 0.6-$-$0.7 mm (computed as RMSE = $\sqrt{MSE}$ from Table 3; MSE reported in mmÂ²), which incorporates this approximation error, remains comparable to manual inter-observer variability, though direct reflex detection or calibration protocols should be implemented in future work. Second, the validation approach for CER measurements relied on inter-algorithm comparison, as no standardized manual measurement protocols exist for this computational metric. While the high segmentation accuracy (Table 2, mIoU > 0.92) and consistent results across different architectures support the geometric validity of our measurements, future studies incorporating 3D imaging or independent validation methods would strengthen clinical confidence in CER quantification. Lastly, this focus on the practical implementation of an integrated system also informed the scope of our evaluation, which was centered on validating the platform as a whole rather than performing a comprehensive benchmark of the segmentation model itself. Consequently, a direct performance comparison against a wide range of other state-of-the-art models was not conducted and remains a direction for future work.

In conclusion, this study proposes a comprehensive system for automated eyelid analysis quantification in clinical settings, incorporating an imaging device and a deep learning-based eye segmentation algorithm. The imaging device utilizes an infrared camera to accurately capture the position of the pupil, and the SegFormer model enables precise segmentation, demonstrating reliable MRD and CER measurements. It is expected that the proposed system will be widely adopted in clinical applications requiring detailed and quantitative measurements of eye region indicators.

## Supplementary Information

Below is the link to the electronic supplementary material.

Supplementary file 1 (pdf 1997 KB)

## Data Availability

The clinical datasets used in this study are not publicly available due to patient privacy and confidentiality restrictions.

## Code availability

Available at: https://github.com/dusrb37/MRD-CER-Analysis.
