# Supplementary material for: A comprehensive system for eyelid analysis using deep learning: automated measurement of eyelid position and corneal exposure
Source: Biomed Eng Lett. 2026 Jan 9;16(3):747–58. doi: 10.1007/s13534-025-00546-9 (PMC13129012; doi:10.1007/s13534-025-00546-9)
Supplement: Supplementary file 1 — (pdf 1997 KB) [file 13534_2025_546_MOESM1_ESM.pdf]

Supplementary Information

Table S1 IR imaging system specifications.

Category	Parameter	Value	Notes
**LED Illuminator**			
	Model	MS-IR510U	Moksan Electronics
	Peak wavelength	850 nm	@ TA=25°C
	Spectral width (FWHM)	˜40 nm	@ 50% intensity
	Radiant intensity	85–110 mW/sr	Manufacturer datasheet
	Viewing angle	±10°	Half-intensity angle
	Forward voltage	1.8 V (typ)	@ IF=50 mA
	Forward current (max)	50 mA	Operating condition
**Image Acquisition**			
	Frame rate	30 fps	Video acquisition
	Processing resolution	256 × 256 pixels	Down-sampled
**Geometry & Alignment**			
	Subject distance	50 cm	Fixed positioning
	Head stabilization	Chinrest + forehead support	-
	Alignment method	Cross-shaped laser	Frankfort horizontal plane
**Calibration**			
	Method	Reference marker-based	No geometric calibration
	Marker specification	5 mm diameter	Circular fiducials
	Scale calculation	Session-wise median	Patient-specific $s_{mm/pixel}$
**Safety**			
	Standard	IEC 62471 compliant	Continuous NIR operation

FWHM, full width at half maximum; NIR, near-infrared.

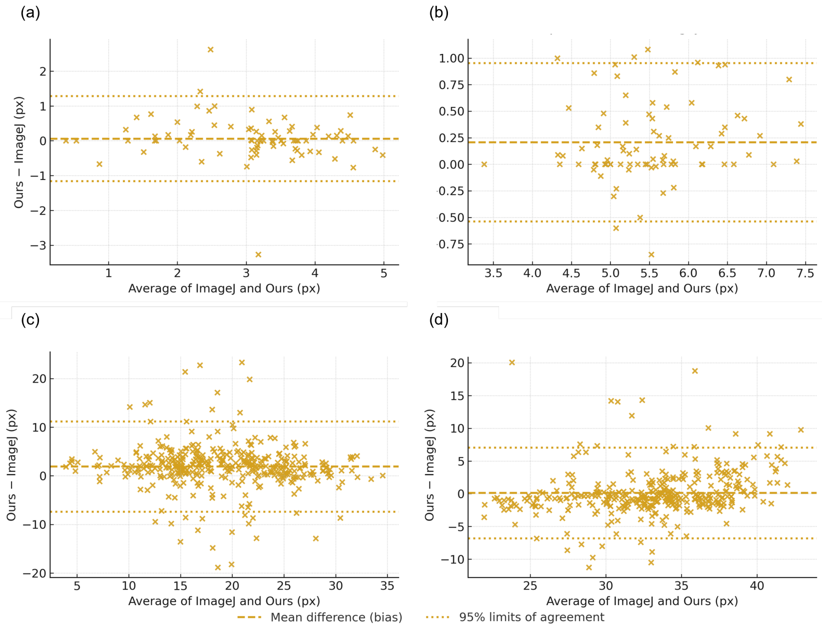

Fig. S1 Bland-Altman analysis for MRD comparing automated and frame-level manual annotations. (a,b) Internal validation for MRD1 and MRD2. (c,d) External validation for MRD1 and MRD2.

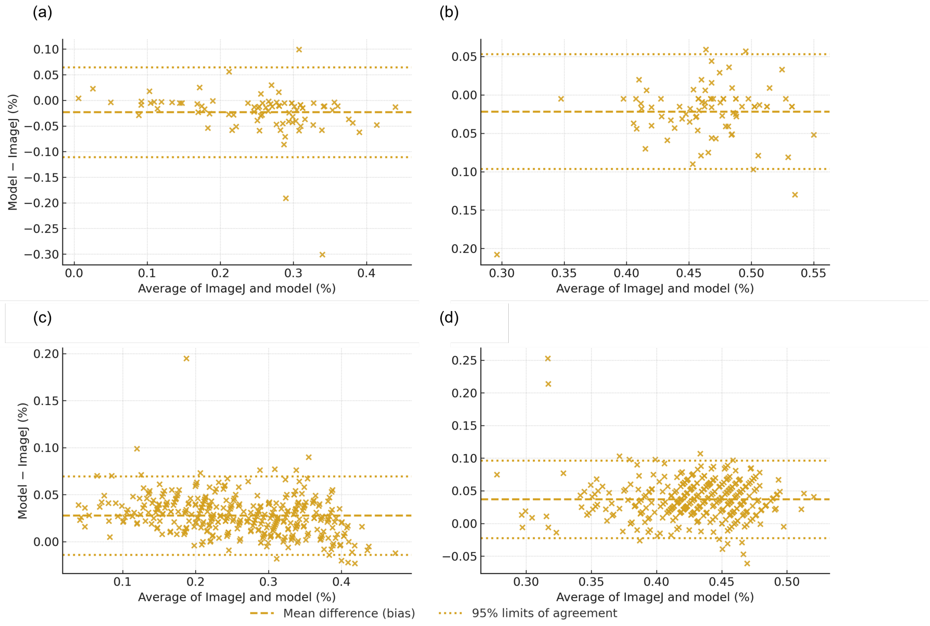

Fig. S2 Bland-Altman analysis for CER comparing automated and frame-level manual annotations. (a,b) Internal validation for upper and lower CER. (c,d) External validation for upper and lower CER.
